# Response to the Comment on Our Article Titled: “Perspective on Antibiotic Resistance in Bangladesh: A Critical yet Overlooked Public Health Crisis”

**DOI:** 10.1002/hsr2.70541

**Published:** 2025-03-04

**Authors:** Md. Mahadi Hassan, Noushin Nohor

**Affiliations:** ^1^ Department of Public Health and Informatics Jahangirnagar University Dhaka Bangladesh; ^2^ aiHealth Lab Center for Health Innovation, Research, Action, and Learning – Bangladesh (CHIRAL Bangladesh) Dhaka Bangladesh; ^3^ Population Health Studies Division Center for Health Innovation, Research, Action, and Learning – Bangladesh (CHIRAL Bangladesh) Dhaka Bangladesh

Dear Editor,

We thank Dr. Jeff Clyde G. Corpuz for his insightful comment, **“Antibiotic Resistance in the Philippines: A Public Health Crisis and Call for Urgent Action,”** on our article [1] which draws critical parallels between the antibiotic resistance (AR) challenges in the Philippines and Bangladesh. We appreciate the emphasis on shared drivers of AR, such as self‐medication, agricultural misuse, and fragmented regulatory frameworks, and fully endorse the call for multisectoral, “One Health” interventions. This correspondence seeks to amplify the discussion by highlighting opportunities for cross‐country collaboration and expanding on actionable strategies to address AR in low‐ and middle‐income countries (LMICs). The Philippines and Bangladesh indeed face strikingly similar AR landscapes, as underscored by Dr. Corpuz. Both nations grapple with high rates of antibiotic misuse, limited public awareness, and systemic gaps in surveillance and stewardship. The Philippines' ongoing efforts to enforce prescription‐only antibiotic access and promote antimicrobial stewardship programs could inform Bangladesh's policies. Dr. Corpuz's emphasis on agricultural practices as a key AR driver warrants further attention. In Southeast Asia, the use of antibiotics in livestock farming remains a critical yet under‐regulated issue. Collaboration between Bangladesh and the Philippines could explore region‐specific alternative solutions for tackling AR, such as probiotics or phage therapy. While national interventions are vital, transnational cooperation is equally crucial. We propose establishing a “Southeast Asia AMR Consortium” to facilitate data sharing, harmonize surveillance protocols, and advocate for unified regulatory standards. Such a platform could accelerate the implementation of the WHO's Global Action Plan on AMR [2], particularly in LMICs where resource constraints hinder progress. Additionally, integrating behavioral science into public education campaigns—as piloted in Bangladesh's community‐led initiatives—could enhance the Philippines' efforts to combat misinformation and promote responsible antibiotic use. Joint pilot programs targeting high‐risk groups (e.g., farmers, and healthcare workers) may yield scalable, culturally adaptive strategies.

Finally, we echo Dr. Corpuz's advocacy for policy reforms but stress the need for innovation tailored to local contexts. For instance, digital health tools (e.g., mobile apps for prescription tracking or telemedicine consultations) could address systemic gaps in rural healthcare access while curbing self‐medication. Similarly, partnerships with academic institutions could foster low‐cost diagnostic technologies to reduce empiric antibiotic prescribing. AR is a borderless threat demanding borderless solutions. By leveraging shared experiences and fostering regional collaboration, the Philippines, Bangladesh, and other LMICs can transform challenges into opportunities for innovation. We urge stakeholders to prioritize cross‐country knowledge exchange, invest in context‐driven research, and amplify advocacy for sustainable policies. Only through collective action can we safeguard the efficacy of antibiotics for future generations.

## Author Contributions

**Md. Mahadi Hassan:** conceptualization, writing – original draft, writing – review and editing. **Noushin Nohor:** conceptualization, writing – original draft, writing – review and editing.

## Conflicts of Interest

The authors declare no conflicts of interest.

## Transparency Statement

The lead authors, Md. Mahadi Hassan and Noushin Nohor affirm that this manuscript is an honest, accurate, and transparent account of the study being reported; that no important aspects of the study have been omitted; and that any discrepancies from the study as planned (and, if relevant, registered) have been explained.

## Data Availability

The authors have nothing to report.
